# O-arm-Assisted Balloon Kyphoplasty for the Treatment of Vertebral Compression Fractures: A Retrospective Study

**DOI:** 10.7759/cureus.85046

**Published:** 2025-05-29

**Authors:** Khalil Makareous Laham, Mamoun Ahmed, Ahmad Kenan Jawhar, Robert Lucaciu, Martin Scholz

**Affiliations:** 1 Department of Neurosurgery, Sana Hospital Duisburg, Duisburg, DEU

**Keywords:** balloon kyphoplasty, cement leakage, o-arm navigation, radiation exposure, vertebral compression fracture (vcf)

## Abstract

Introduction: Vertebral compression fractures (VCFs) are a common manifestation of osteoporosis and are getting more common as the world's population ages. Over the years, balloon kyphoplasty (BK) has established itself as a popular treatment modality for osteoporotic VCFs. O-arm-guided navigation is one of the innovative techniques that improve safety and precision during spine surgeries. This study aims to evaluate the accuracy and safety of O-arm-assisted BK (O-BK) in the management of VCFs. Material and methods: This retrospective, single-center analysis includes patients who underwent O-BK at our institution between 2018 and 2023. The main imaging modality was the O-arm O2 Surgical Imaging System (Medtronic, Dublin, Ireland). The inclusion criteria consisted of patients with acute, painful traumatic or osteoporotic VCFs involving a maximum of three lumbar or thoracic levels and presenting with localized back pain without accompanying radicular symptoms. Patients with concomitant degenerative spinal diseases relevant to their symptoms or with unstable fractures were excluded from the study. The main objectives were to evaluate operation duration, radiation exposure, and the rate of cement leakage.

Results: A total of 74 patients with 102 vertebral levels were treated. The mean age of patients was 75.4 years, with the majority being females (71.6%, 53/74). Osteoporotic fractures were the primary diagnosis in 78.4% (58/74) of patients. The mean operation time was 63.01 minutes. The mean fluoroscopy time was 36.7 seconds. The overall mean effective dose was 9.22 mSv. Cement leakages occurred in 33.3% (34/102) of treated levels, with an intraspinal leakage rate of 3.9% (4/102). No patient experienced symptomatic leakages. Conclusions: O-BK appears to be a safe and accurate minimally invasive procedure for treating VCFs, especially those associated with osteoporosis. In addition to the controlled radiation dose, the procedure's safety is demonstrated by the low incidence of intraspinal cement leakages and the absence of neurological deficits. O-arm technology's enhanced imaging and navigation precision, which enables accurate and secure treatments, is a significant advantage.

## Introduction

Vertebral compression fractures (VCFs) commonly result as a complication of osteoporosis, a condition that is characterized by diminished bone density and quality [1,2]. Osteoporosis represents a major global health challenge, with its prevalence rising as populations age. Even though it is widespread, there are significant deficiencies in diagnosis and treatment. VCFs can result in intense pain, disability, and diminished mobility, which may degrade the quality of life and heighten the risk of complications such as pneumonia, especially among older adults [3]. To restore functionality, reduce the risk of fractures, and enhance overall quality of life, timely intervention and rehabilitation are essential.

The treatment strategies for VCFS have undergone significant changes over time. Balloon kyphoplasty (BK), a commonly used intervention, has shown superior outcomes compared to conservative management, especially for osteoporotic fractures, due to its short- and long-term capacity to reduce pain and restore vertebral body height [4-6].

Furthermore, the efficacy and safety profile of spine surgeries has been significantly improved in recent years with the incorporation of O-arm-assisted navigation, which offers surgeons real-time, high-resolution intraoperative image guidance during procedures [7,8].

Despite these procedural advances, a lack of research remains on the utility of O-arm in BK and its evaluation in comparison to conventional methods. The majority of the literature focuses on other spine procedures, such as the placement of pedicle screws. The utilization of O-arm-assisted BK (O-BK), particularly in the analysis of intraoperative metrics such as administered radiation dose, cement leakage, and operation duration, remains understudied, a gap that our study seeks to address.

This study presents a retrospective, single-center analysis evaluating key intraoperative metrics during O-BK osteotomies performed for osteoporotic VCFs.

## Materials and methods

Study design

This is a retrospective single-center study conducted at Sana Kliniken Duisburg, Duisburg. The data were collected from the electronic data of all patients who underwent O-BK between April 2018 and August 2023. The O-arm O2 Surgical Imaging System (Medtronic, Dublin, Ireland) was used as the imaging modality.

Inclusion and exclusion criteria

Patients with acute, painful traumatic or fragility-related osteoporotic VCFs, limited to a maximum of three affected thoracic or lumbar vertebral levels, were included. All patients reported localized back pain without accompanying radicular symptoms. These patients underwent O-BK at our clinic during the study period. Patients with concomitant degenerative spinal diseases relevant to their symptoms or with unstable fractures were excluded from the study. Patients with incomplete datasets were also excluded.

Measured parameters

Patients’ demographics included age (in years) and sex (male or female). The number of treated vertebral levels, the spinal region (thoracic or lumbar), the total operative time (from skin incision to closure, measured in minutes), and the rate of cement leakage, which was categorized as intraspinal, intradiscal, or paravertebral, were recorded and analyzed. Radiation exposure was assessed for both 2D fluoroscopy and 3D imaging (O-arm). For 2D fluoroscopy, the recorded parameters included fluoroscopy time (in seconds) and dose area product (DAP) measured in Gy·cm². For 3D imaging, the dose length product (DLP) in mGy·cm and the computed tomography dose index (CTDI) in mGy were documented. Radiation exposure data were automatically recorded and displayed in the O-arm system report at the end of each procedure.

Based on established guidelines, conversion factors were used to calculate the effective radiation dose (ED, mSv). According to European Commission Report No. 180 [9], the effective dose for 2D fluoroscopy was calculated using conversion factors f (in mSv/(Gy·cm²)), with values of 0.19 mSv/(Gy·cm²) for the thoracic spine and 0.26 mSv/(Gy·cm²) for the lumbar spine. The effective dose was then calculated using the formula:

\[ E\,(\text{mSv}) = \text{DAP}\,(\text{Gy} \cdot \text{cm}^2) \times f \]

The effective dose for 3D imaging was calculated from the DLP in accordance with the European Guidelines for Multislice Computed Tomography [10] and Dosimetry Report for the O-arm O2 Surgical Imaging System [11] using a region-specific conversion coefficient k (in mSv/(mGy·cm)). The k values used were 0.014 mSv/(mGy·cm) for the thoracic spine and 0.015 mSv/(mGy·cm) for the lumbar spine. Here was the effective dose calculated using the formula:

\[ E\,(\text{mSv}) = \text{DLP}\,(\text{mGy} \cdot \text{cm}) \times k \]

Surgical procedure

**Figure 1:**
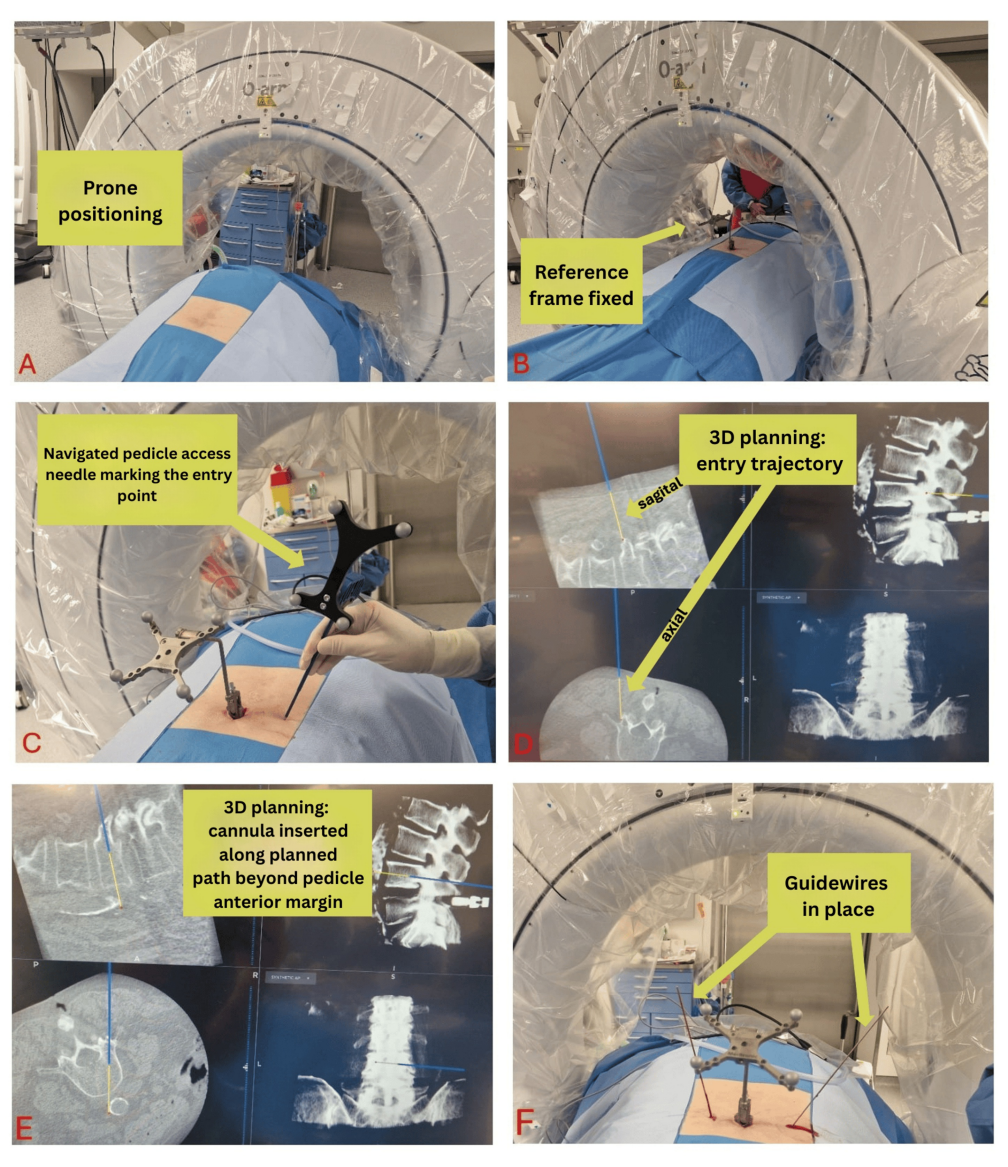
Surgical procedure (A) Patient positioning in the prone position under general anaesthesia. (B) Patient preparation for the initial 3D scan using the O-arm after fixation of the navigation reference. (C-D) Determination of the optimal skin entry point using a virtual trajectory with a pedicle access needle, (E) Insertion of the cannula along the planned path, surpassing the anterior border of the pedicle, (F) Placement of guidewires after removal of the cannula. 3D: three dimensional

The procedure is performed under general anesthesia, with the patient positioned in a prone position (Figure 1A). The fracture level is localized using O-arm fluoroscopy (O-arm by Medtronic) as the imaging modality. A midline skin incision is made at the level or one level below the fracture. The navigation reference frame is then attached to the spinous process. A 3D scan is performed using the O-arm (Figure 1B), and the 3D images are transferred to the StealthStation S8 Surgical Navigation System (Medtronic, Dublin, Ireland) for navigation. The Medtronic Navigated Pedicle Access Kit, consisting of a cannula and a pedicle access needle, is used to determine the optimal entry point on the skin using a virtual extension (Figure 1C). A small puncture skin incision is made, and the navigated cannula is guided percutaneously to the bone. The cannula is then hammered transpedicularly along the indicated route until it surpasses the anterior border of the pedicle (Figure 1D). The pedicle access needle is removed, and a guidewire is inserted through the cannula, which is then removed. The procedure is repeated on the contralateral side (Figure 1E). The subsequent steps, including inserting the introducer, drilling, balloon inflation, and cement application, are performed under O-arm fluoroscopic control, similar to a standard kyphoplasty procedure.

Statistical analysis

Statistical analysis was performed using SPSS Statistics version 28 (IBM Corp. Released 2021. IBM SPSS Statistics for Windows, Version 28.0. Armonk, NY: IBM Corp.). For normally distributed continuous variables, parametric tests (t-test and ANOVA) were used. In contrast, non-parametric tests (Mann-Whitney U test and Kruskal-Wallis test) were applied for non-normally distributed data. Categorical variables were analyzed using the chi-square test or Fisher’s exact test, as appropriate. A p-value of <0.05 was considered statistically significant.

## Results

Patient characteristics

A total of 74 patients underwent O-BK involving 102 vertebral levels. The mean age was 75.4 ± 10.3 years. Almost three-quarters of the study population (72%) were female. Fifty-eight patients (78%) had osteoporotic fractures, whereas 16 patients (22%) had traumatic fractures.

Surgical characteristics

**Figure 2:**
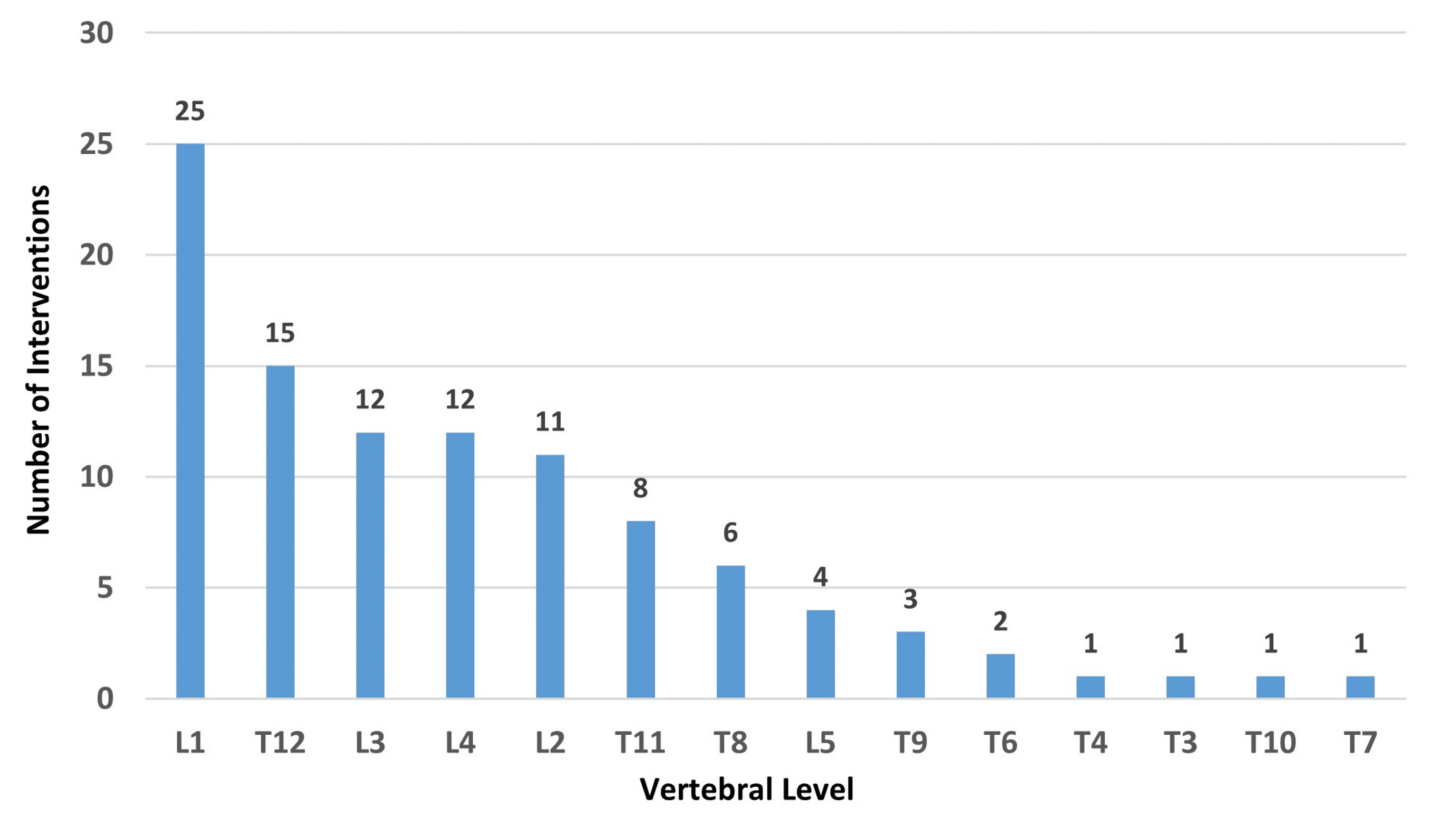
Number of O-BK procedures per vertebral level (n=102) T: thoracic vertebra, L: lumbar vertebra, O-BK: O-arm-assisted balloon kyphoplasty

Two-thirds of the study population (68%) were operated on for one VCF, whereas 27% of the population received O-BK on two VCFs simultaneously. Only 5% of the study sample received therapy on three VCFs in one sitting. In total, 64 lumbar vertebrae and 38 thoracic vertebrae were operated on. The highest affected thoracic vertebra was T3, and the lowest lumbar vertebra was L5. The most frequently operated vertebral levels in this study were L1 (24.5%), T12 (13.7%), L3 (11.8%), L4 (11.8%), and L2 (10.8%). The remaining levels were operated on less frequently, each contributing a smaller proportion to the total cases (Figure 2).

**Figure 3:**
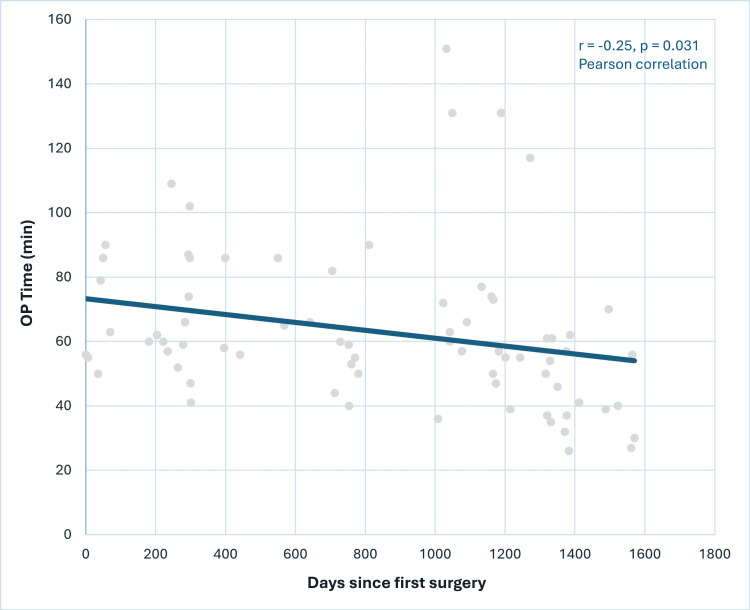
Trend of operative time over study period A significant negative correlation was observed between operative duration and surgery date (r=-0.25, p=0.031, Pearson correlation). OP: operative

The mean operative time was 63.0 ± 24.1 minutes, and the mean operative time per level was 45.7 minutes. A significant negative correlation was observed between operative time and surgery date (r=-0.25, p=0.031), indicating a reduction in operative time over the study period (Figure 3), which suggests a significant improvement in efficiency.

Radiation exposure

The mean 2D fluoroscopy time was 35.9 ± 33.3 seconds. The mean DAP was 10.3 ± 8.8 Gy/cm². In 3D imaging, the mean DLP was 463.9 ± 247.5 mGy·cm, while the mean CTDI was 28.9 ± 15.7 mGy. The mean effective dose in 2D and 3D imaging was 2.4 ± 2.1 mSv and 6.8 ± 3.6 mSv, respectively, while the overall mean effective dose across all imaging modalities was 9.2 ± 4.3 mSv.

The cumulative fluoroscopy time increased understandably with the number of vertebral levels treated. Patients undergoing single-level kyphoplasty had a mean fluoroscopy time of 29.6 ± 26.2 seconds, compared to 43.3 ± 36.4 seconds for two levels and 75.8 ± 53.4 seconds for three levels. However, this trend did not reach statistical significance (p=0.100). There was no significant change in fluoroscopy time throughout the study period (p=0.524). Moreover, there was no significant change in fluoroscopy time in relation to thoracic and lumbar levels (p=0.701).

Cement leakage

The mean cement volume applied across all cases was 3.7 ± 1.0 ml. The overall cement leakage rate observed in this study was 33.3% (34/102 cases). Most commonly observed was the paravertebral leakage in 19.6% (20/102), followed by intradiscal leakage comprising 15.7% (16/102), whereas intraspinal leakage was observed only in 3.9% (4/102) of the cases. There were no cement-related neurological or systemic complications. There was no need for intraoperative revision due to cement leakage.

There was no statistically significant association between cement leakage and the amount of cement injected (mean 3.7 ± 1.0 ml; p=0.520). Similarly, no significant difference in leakage rates was found between thoracic and lumbar vertebrae (p=1.0). Over time, there was no significant reduction in leakage rates throughout the study period (p=0.327). The overall results of the study are summarized in Table 1.

**Table 1:** Summary of the results 2D: two dimensional, 3D: three dimensional, DAP: dose area product, DLP: dose length product, CTDI: computed tomography dose index

Number of patients	74
Number of treated vertebrae	102
Gender (f/m)	53/21
Age (y)	75.40 ± 10.30
Pathology n (%)	
Osteoporotic	58 (78.4%)
Traumatic	16 (21.6%)
Treated vertebrae/patient n (%)	
1 level	50 (67.6%)
2 levels	20 (27%)
3 levels	4 (5.4%)
Fracture location n	
Thoracic	38
Lumbar	64
Operative time (min)	63.01 ± 24.14
Operative time per level (min)	45.72
2D fluoroscopy time (sec)	35.89 ± 33.27
DAP (Gy.cm^2^)	10.341 ± 8.816
DLP (mGy.cm)	463.87 ± 247.49
CTDI (mGy)	28.92 ± 15.71
2D effective dose (mSv)	2.44 ± 2.16
3D effective dose (mSv)	6.78 ± 3.62
Overall effective dose (mSv)	9.22 ± 4.26
Cement volume (ml)	3.73 ± 1.03
Cement leakage n (%)	34 (33.3%)
Intraspinal	4 (3.9%)
Intradiscal	16 (15.7%)
Paravertebral	20 (19.6%)

## Discussion

O-arm navigation has become increasingly utilized in spine surgery, enhancing the precision and safety of various procedures. Pedicle screw fixation is one of its most popular applications, as navigation-assisted placement has been shown to improve accuracy and reduce the likelihood of malposition [7,12-14]. Additionally, in transarticular screw fixation for atlantoaxial instability and anterior cervical screw fixation for odontoid fractures, O-arm guidance has demonstrated superior accuracy compared to conventional techniques [15,16]. Moreover, O-arm navigation-guided biopsy offers enough tissue from a precise location to diagnose spine pathologies [17]. These advantages have also been utilized in vertebroplasty and BK, as O-arm navigation improves needle placement accuracy and enhances control over cement injection, potentially reducing cement leakage and other complications.

Regarding operative time, Schils et al., who were the first to describe O-arm navigated kyphoplasty, reported in their preliminary series an operative time of 41 minutes, which was reduced in the final series to 38 minutes [18,19]. Dohm et al. revealed that BK and vertebroplasty had mean operative times of 40 and 32 minutes, respectively [20]. Furthermore, Ji et al. compared single-level surgery time for the O-arm and C-arm groups, which were 26.4 ± 5.4 minutes and 38.1 ± 6.6 minutes, respectively [21]. On the contrary, our study demonstrated a longer operative time compared to both conventional C-arm-guided kyphoplasty (Dohm et al., Ji et al.) and other O-arm-guided procedures (Schils, Ji et al.). This difference may be attributed to variations in workflow. A detailed comparison of operative durations between our study and previous literature is provided in Table 2.

**Table 2:** Operative duration in our study compared to published literature

Study	Method	Mean operative duration (minutes)	Sample size (n)
Our study	O-arm	63.01 ± 24.14	74
Dohm et al. (2014) [20]	C-arm	40.0	191
Ji et al. (2025) [21]	O-arm	26.43 ± 5.42	28
Ji et al. (2025) [21]	C-arm	38.17 ± 6.68	30
Schils (2011) [19]	O-arm	38 (22-95)	54

The extended operating duration observed in our cohort may have been partly attributed to variations in surgical expertise and familiarity with the O-arm system. Furthermore, the use of O-arm navigation adds several procedural steps, including trajectory planning, image acquisition, and real-time intraoperative adjustments, which may initially result in longer operating times, especially during the early stages of adoption. Our study's results, which showed a decreasing operative duration over time (r=-0.25, p=0.031) (Figure 3), confirm this by indicating a continuous learning curve effect.

Radiation exposure remains a substantial safety concern in BK, since fluoroscopic guidance is usually necessary for placing the cannula and applying the cement. With traditional C-arm fluoroscopy, there is often a long duration of continuous intraoperative exposure for both patients and surgical staff. On the other hand, O-arm navigation has the benefit of providing an initial 3D scan. This enables trajectory planning and instrumentation without the need for ongoing fluoroscopy. Notably, the 3D scan is conducted with all surgical staff outside the operating room, which eliminates their exposure during this phase.

Radiation exposure in fluoroscopically guided BK was reported in a study by Perisinakis et al. with a mean fluoroscopy time of 606 ± 132 seconds [22], substantially higher than in our study (35.9 ± 33.3 seconds). Moreover, Wojdyn et al. compared O-arm and C-arm guided vertebroplasty, with a primary focus on fluoroscopic exposure to the surgical staff. DAP was significantly lower in the O-arm group (9.1 ± 4.1 Gy/cm²) than in the C-arm group (17.2 ± 8.8 Gy/cm²) [23]. Our DAP of 10.3 ± 8.8 Gy/cm² was significantly lower than that of Wojdyn's C-arm group (p=0.0003) and equivalent to their O-arm group (p=0.987). The statistics demonstrate that by avoiding continuous fluoroscopy during needle placement, O-arm navigation significantly lowers the radiation exposure to the surgical staff.

Furthermore, Prod'homme et al. examined comparable radiation metrics in O-arm-guided kyphoplasty utilizing an all-in-one 2D/3D Surgivisio system [24]. Our 3D effective dose was significantly lower (6.7 ± 3.6 mSv compared to 9.2 ± 6.7 mSv, p=0.013), as was the total effective dose (9.2 ± 4.2 mSv compared to 11.4 ± 7.3 mSv, p=0.035). The fluoroscopy time reported for their O-arm group was 26 ± 15.4 seconds, which was significantly lower than the time recorded in our study (35.9 ± 33.3 seconds, p=0.024). However, the DAP values were comparable (10.2 ± 9.3 Gy·cm² vs. 10.3 ± 8.8 Gy·cm², p=0.999). This could be due to variations in O-arm scan protocols or dose modulation methods between institutions. Moreover, variability in fluoroscopy time seen in our cohort is likely due to variations in case complexity, such as multilevel involvement, patient body habitus (e.g., obesity), and anatomical difficulties.

Our results fall within this range and suggest that, even when both 2D and 3D imaging components are included, O-arm navigation does not necessarily result in increased overall radiation exposure and stays under the threshold for cancer induction [25]. Table 3 presents a comparative overview between our study and Prod'homme et al.'s study.

**Table 3:** Comparison of radiation parameters with Prod’homme et al. 2D: two dimensional, 3D: three dimensional, DAP: dose area product

Parameter	Our study	Prod’homme et al. (2022) [24]	p-value
Fluoroscopy time (sec)	35.89 ± 33.27	25.99 ± 15.35	0.024
2D effective dose (mSv)	2.44 ± 2.16	2.24 ± 1.80	0.557
3D effective dose (mSv)	6.78 ± 3.62	9.22 ± 6.78	0.013
Total effective dose (mSv)	9.22 ± 4.26	11.47 ± 7.32	0.035
DAP (Gy/cm²)	10.34 ± 8.82	10.22 ± 9.33	0.999

Cement leakage is one of the most challenging complications of BK. In the worst-case scenario, it may result in neurological deficits, necessitate revision surgery, and increase procedural costs, underscoring the importance of early detection and prevention. In a study by Riesner et al., which compared BK and radiofrequency kyphoplasty (RFK) in 100 patients with 176 vertebral fractures, the cement leakage rate was 63.9% in the RFK group and 60.8% in the BK group, with most of these leaks occurring intradiscally [26]. On the contrary, Zhan et al. reported a leakage rate of 18% [27]. Therefore, analyzing this complication in clinical studies is essential to assess the safety profile of the procedure.

In our study, the overall leakage rate was 33.3%, including 3.9% intraspinal, 15.7% intradiscal, and 19.6% paravertebral. None of the leakages resulted in neurological deficits. In the multivariate analysis performed, there was no significant difference between cement leakage in thoracic and lumbar vertebrae. Surgical experience, cement viscosity, and cement application technique may explain the discrepancies in cement leakage rates compared to other studies.

This underscores a key advantage of O-arm-navigated kyphoplasty, which is the ability to perform an intraoperative 3D scan in cases of suspected leakage. This enables immediate evaluation of cement distribution and, if necessary, timely intervention during the same surgical session. As navigation significantly reduces the risk of needle malposition [21], the importance of optimizing cement application techniques should be taken into consideration.

Considering the learning curve associated with O-arm navigation is also justifiable, as it may impact not only efficiency but also procedural safety, especially in centers with limited experience. In such environments, the lack of standardized workflows and unfamiliarity with intraoperative 3D imaging may lead to increased complication rates, including cement leakage. This is particularly concerning when it involves intraspinal or paravertebral spaces, as it can result in significant long-term consequences, such as neurological deficits, the need for revision surgery, and increased healthcare costs. The results highlight the significance of organized training and workflow standardization in reducing safety risks associated with the learning curve in facilities newly implementing O-arm navigation.

Clinical implications

Our findings support the utility of O-BK as a safe and precise technique. Despite a slightly longer operative duration, the benefits of reduced radiation exposure for surgical staff, combined with acceptable cement leakage rates and no clinical sequelae, affirm its role in treating VCFs. As centers adopt O-arm guidance, proper training and protocol optimization are essential to maximize safety and efficiency.

Study limitations

The study is retrospective, which limits the amount of evidence and may introduce bias. Additionally, there are no assessments of clinical or radiological outcomes, and no follow-up data are available. This limits the ability to assess the procedure's long-term efficacy. Variations in surgeon experience and workflow preferences may have influenced operation parameters such as procedure duration, cement application, and radiation exposure. More prospective studies should be conducted to assess clinical and radiological outcomes in standardized settings.

## Conclusions

O-BK is a safe and precise, minimally invasive treatment option for VCFs, especially those associated with osteoporosis. The procedure's safety is demonstrated by the low rate of intraspinal cement leakage and the lack of symptomatic cases. Additionally, the technique's overall safety is supported by the measured radiation exposure. Procedural reliability can be further improved through ongoing efforts to refine cement application techniques and imaging protocols, thereby reducing cement leakage rates and shortening operative durations.
